# Assessment of the burden of Giardia infection and its associated risk factors among children resident in internally displaced persons camps in Mogadishu, Somalia: a cross-sectional study

**DOI:** 10.1093/inthealth/ihaf034

**Published:** 2025-04-07

**Authors:** Bashiru Garba, Najib Isse Dirie, Yushau Umar, Abdikani Omar Salah, Ahmed Abdirahim Hussien, Ikram Abdirahman Mohamud Alasow, Hodo Aideed Asowe, Fartun Abdullahi Hassan Orey, Jihaan Hassan, Jamal Hassan Mohamoud, Mohamed Hussein Adam, Mohamed Adam Mahamud, Ibrahim Abdullahi Mohamed, Abdullahi Abdirahman Omar, Mohamed Mustaf Ahmed

**Affiliations:** Department of Public Health, Faculty of Medicine and Health Sciences, SIMAD University, Jidka Warshadaha, 2526, Mogadishu, Somalia; Department of Veterinary Public Health and Preventive Medicine, Faculty of Veterinary Medicine, Usmanu Danfodiyo University, Sokoto, 840212, Sokoto State, Nigeria; Department of Urology, Dr. Sumait Hospital, Faculty of Medicine and Health Sciences, SIMAD University, Jidka Warshadaha, 2526, Mogadishu, Somalia; Division of Veterinary Public Health and Preventive Medicine, National Veterinary Research Institute Vom, 800101, Jos, Plateau State, Nigeria; Department of Microbiology and Laboratory Sciences, Faculty of Medicine and Health Sciences, SIMAD University, Jidka Warshadaha, 2526, Mogadishu, Somalia; Department of Microbiology and Laboratory Sciences, Faculty of Medicine and Health Sciences, SIMAD University, Jidka Warshadaha, 2526, Mogadishu, Somalia; Department of Microbiology and Laboratory Sciences, Faculty of Medicine and Health Sciences, SIMAD University, Jidka Warshadaha, 2526, Mogadishu, Somalia; Department of Nursing and Midwifery Science, Faculty of Medicine and Health Sciences, SIMAD University, Jidka Warshadaha, 2526, Mogadishu, Somalia; Department of Pediatrics and Child Health, Dr. Sumait Hospital, Faculty of Medicine and Health Sciences, SIMAD University, Jidka Warshadaha, 2526, Mogadishu, Somalia; Department of Pediatrics and Child Health, Dr. Sumait Hospital, Faculty of Medicine and Health Sciences, SIMAD University, Jidka Warshadaha, 2526, Mogadishu, Somalia; Department of Public Health, Faculty of Medicine and Health Sciences, SIMAD University, Jidka Warshadaha, 2526, Mogadishu, Somalia; Department of Public Health, Faculty of Medicine and Health Sciences, SIMAD University, Jidka Warshadaha, 2526, Mogadishu, Somalia; Department of Microbiology and Laboratory Sciences, Faculty of Medicine and Health Sciences, SIMAD University, Jidka Warshadaha, 2526, Mogadishu, Somalia; Dr. Sumait Hospitals, Faculty of Medicine and Health Sciences, SIMAD University, Mogadishu, Somalia; Dr. Sumait Hospitals, Faculty of Medicine and Health Sciences, SIMAD University, Mogadishu, Somalia; Faculty of Medicine and Health Sciences, SIMAD University, Jidka Warshadaha, 2526, Mogadishu, Somalia

**Keywords:** deworming, hygiene, intestinal parasitic infections, Somalia, under-5 children, WASH

## Abstract

**Background:**

This study was conducted to determine the prevalence of *Giardia duodenalis* infection and identify potential risk factors in a healthy population of children living in internally displaced persons (IDP) camps, in Mogadishu, Somalia.

**Methods:**

A community-based cross-sectional study was conducted among children living in Deyniile and Kahda IDP settlements, Mogadishu. A total of 334 children were randomly selected for stool sample collection and a questionnaire was administered. Giardia infection was diagnosed by antigen detection using a rapid detection kit.

**Results:**

The results showed that 32 of the 334 children were positive, giving an overall prevalence of 9.6%. We also found that 196 (58.7%) of the children did not wear footwear, and that a majority of the members of the household (308; 92.2%) practiced open defecation. The regression analysis revealed that children aged <5 y (p=0.002), households in the Kahda IDP camp (p=0.019) and families with >5 members in their households (p=0.034) all have a significantly higher risk of becoming infected with Giardia.

**Conclusion:**

The study found that giardiasis persists in the IDP camps. Many of the risk factors were associated with giardiasis, highlighting the significance of parents' education and sanitation conditions in the children's well-being.

## Introduction

Giardiasis, an intestinal parasitic infection, is a cosmopolitan parasite mostly associated with diarrheal disease throughout the world.^1^ The disease is spread through contaminated food, water or surfaces, particularly in resource-limited countries.^1^ Common clinical manifestations include diarrhea, abdominal cramps, nausea and fatigue. The parasite thrives in areas with poor sanitation, due to the contamination of water and food with fecal matter. The lack of proper sewage and waste disposal systems, as well as clean drinking water, along with improper hygiene practices, create an environment where the parasite can easily spread, leading to higher infection rates in these regions.^2,3^

The burden of giardiasis is significantly higher in developing countries compared with industrialized countries because of inadequate access to clean water, poor sanitation and a lack of hygiene awareness.^4,5^ It is one of the leading causes of diarrheal illness, and particularly affects children.^6^ Contaminated water sources and improper food handling increase the transmission of Giardia, contributing to widespread infections and public health challenges. Recently, a systematic review and meta-analysis of *Giardia duodenalis* indicated a pooled prevalence of 18.3% among African children.^7^ In addition to the limited access to clean water, inadequate sanitation and poor hygiene practices that characterize most African countries, overcrowded living conditions and a lack of effective water treatment systems significantly contribute to the widespread transmission of Giardia parasites, especially in rural and low-income communities across the continent.^8^ In addition, the high burden of Giardia in Africa and other developing countries is attributed to a number of socioeconomic factors, including poverty, inadequate healthcare infrastructure and overcrowding in urban slums and rural areas, as well as low education levels.^8^

Giardiasis is a significant public health concern in Somalia due to poor sanitation, limited access to clean water and inadequate healthcare infrastructure.^9^ The disease contributes to morbidity, straining Somalia's fragile healthcare system. A wide range of prevalence has been reported from various studies conducted in different communities, settings and regions, with the lowest prevalence reported being 3.85% from a hospital-based retrospective study in Mogadishu, to as high as 16% among rural children and mothers, 22.1% among children with malnutrition in Banadir region and 60.84% in a 5-y retrospective survey of intestinal parasites in Somalia.^9–11^ In all these, we can appreciate that giardiasis is a significant public health concern in Somalia, particularly among vulnerable populations such as internally displaced persons (IDP) due to poor living conditions. Overcrowding in camps tends to exacerbate the spread of the infection, leading to outbreaks.^12^ Giardiasis can cause severe diarrhea, malnutrition and dehydration, particularly in children, further weakening an already at-risk population.

Although *G. duodenalis* is recognized as a common parasite in humans in Somalia, the actual prevalence of this infection in children residing in IDP settlements remains unknown.

The goal of the current work is to estimate the prevalence of *G. duodenalis* infection and to identify possible risk factors associated with the disease in apparently healthy children living in the Deyniile and Kahda IDP camps, Mogadishu, Somalia. This study among children resident in IDP camps in Mogadishu, Somalia, will guide strategic interventions in order to curb the menace caused by this important public health problem.

## Materials and methods

### Study design and location

**Figure 1.**
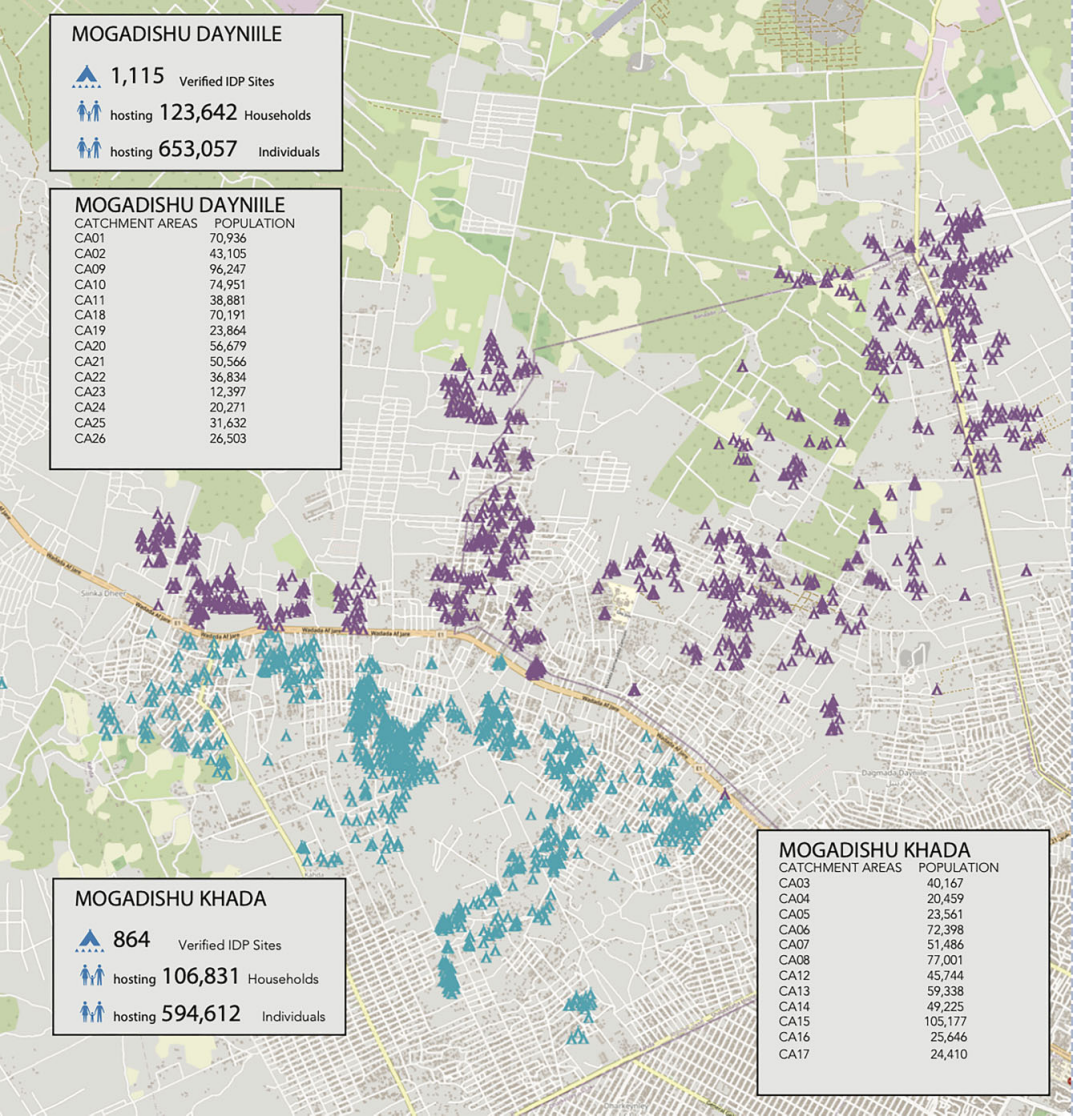
Verified IDP sites in Deyniile and Kahda (CCCM Cluster, Somalia; November 2023). CCCM: Camp Coordination and Camp Management; IDP: internally displaced persons.

This community-based cross-sectional study was conducted in Deyniile and Kahda IDP camps located in the capital city of Mogadishu, Banadir region, Somalia (Figure 1). Mogadishu is home to the largest concentration of IDP in Somalia, who are predominantly members of minority clans displaced from rural areas as a result of conflict and drought.^13^ A recent IDP site verification exercise identified 1979 IDP camps (1115 in Deyniile and 864 in Kahda), hosting a combined total of 230 473 households (1 247 669 individuals).^14^

### Sample size determination

Based on a single population proportion formula, and a reported prevalence of *Giardia lamblia* infection in children of 27.1% in neighboring Ethiopia,^15^ the sample size was calculated as follows:


\begin{equation*}
{\mathrm{N}} = {{\mathrm{Z}}^2}P(1 - P) \div {{\mathrm{e}}^2}
\end{equation*}

At 95% CI, a minimum sample size of 304 was arrived at. However, to compensate for the non-response and non-compliance of the participants, a 10% non-response rate was added to obtain a final sample size of 334.^15^

### Sampling technique

Households within the two camps were selected randomly. Following convenient selection of the first house, subsequently every fifth house was enrolled if it contained children (aged <18 y) and consented. In the event that there were no children, or a household refused to participate, then the preceding house was selected.

### Collection of stool samples and laboratory analysis

Households were systematically selected for both stool and questionnaire data collection. To ensure a representative sample was collected, the first house in each of the camps was conveniently selected, and thereafter every 5th house was chosen. Households were only included if they consisted of residents with at least one child at the time of the visit and provided consent. In total, 334 stool samples were collected into sterile stool collection specimen bottles and transported to the laboratory for processing. Giardia was detected in the stool samples using a commercial Biopanda (UK) *Giardia lamblia* rapid test kit for qualitative detection of the *G. lamblia* antigen in human feces according to the manufacturer's instructions. The kits have a relative sensitivity of 96.2%, relative specificity of 97.8% and accuracy of 97.5%. Briefly, the fecal sample is diluted into the extraction buffer that is supplied with the test and 2–3 drops of the diluted fecal specimen are placed into the cassette and when the extracted specimen comes into contact with the strip, the conjugate migrates with the sample by passive diffusion and the conjugate and sample material come into contact with the anti-Giardia antibody in the T line of the cassette. If the sample contains the *G. lamblia* antigen, the conjugate-antigen complex will bind to the anti-Giardia reagent and a red line will develop. The specimen extract will continue to migrate to encounter a second reagent that binds the migration control conjugate, thereby producing a red control line that confirms the test is working properly. The result is usually visible and interpreted within 15 min.

### Questionnaire tool and study variables

To ensure the accuracy and reliability of the data, face-to-face interviews were conducted with guardians or primary caregivers using a comprehensive questionnaire and observation checklist to assess the sociodemographic, behavioral and environmental risk factors associated with giardiasis using a Google form. The observation checklist and the questionnaire were developed after an extensive literature review.^15–17^ The observation checklist was designed to gather details of environmental factors, such as domestic water sources and storage containers, waste disposal and the hygiene conditions of the households and their surroundings, the type of toilet facilities, as well as ownership of animals (pets or livestock). On the other hand, the questionnaire focused on individual sociodemographic factors (e.g. income, employment status and level of education) and household risk factors (e.g. family size; water, sanitation and hygiene [WASH] behavior).

While *G. lamblia* infection status was used as the dependent variable, the independent variables were grouped into sociodemographic factors and environmental and behavioral risk factors.

### Data analysis

Data exported from the Google form in the form of an Excel spreadsheet were cleaned and coded before being entered into IBM Corp. Released 2023. IBM SPSS Statistics for Windows, Version 27.0 Armonk, NY, USA v. 27 for analysis. All categorical variables were analyzed using descriptive statistics and are expressed as frequencies and percentages, while continuous variables are expressed by the mean and ±SD. Bivariate and multivariate logistic regression was used to assess the association between the dependent variables and independent variables and the OR was used to assess the strength of the association based on p<0.5 (statistical significance). In the bivariate logistic regression model, p<0.25 was considered to be a confounder. Then the confounded factors were entered and analyzed by multivariate logistic regression to measure the strength of the associations, with p≤0.05 considered significantly associated with the prevalence of Giardia infection.^18^

## Results

### Prevalence of Giardia and its relationship with sociodemographic characteristics of the study participants

The majority of the study participants (205; 61.4%) were aged <5 y, while assessment of their education level showed that 193 (57.8%) did not attend any school. However, 138 (41.3%) have Quranic education, with only a paltry total of three (0.9%) having primary education. Evaluation of the age-specific prevalence indicates that most of the infected children were among those children with no schooling (23; 11.9%), followed by children with Quranic education 9 (6.5), while none of the three children with primary level education were positive. As regards parents’ education, the majority reported not attending any school, with totals of 284 (85.0%) and 289 (86.5%) for fathers and mothers, respectively.

*Giardia lamblia* parasite antigen was detected from fresh stool samples collected from the children. According to the rapid Giardia antigen detection test, 32 out of the total of 334 children were found to be positive, giving an overall prevalence of 9.6%, while the majority (90.5%) were negative. The rate for female children was slightly higher with 182 (representing 54.5%). In terms of the gender prevalence, 18 (5.4%) female children and 14 (4.1%) male children were found to be infected with *G. lamblia* infection (Table 1).

**Table 1.** Prevalence of Giardia in relation to participants’ sociodemographic factors

		Status of Giardia infection		
Variable	Category	Negative (%)	Positive (%)	Total (%)
Gender	Male	138 (90.8)	14 (9.2)	152 (45.5)
	Female	164 (90.1)	18 (9.9)	182 (54.5)
Age category	<5 y	176 (85.9)	29 (14.1)	205 (61.4)
	6–10 y	109 (97.3)	3 (2.7)	112 (33.5)
	11–15 y	17 (100)	0 (0.0)	17 (5.1)
Residence	Deyniile	140 (87.0)	21 (13.0)	161 (48.2)
	Kahda	162 (93.6)	11 (6.4)	173 (51.8)
Level of child’s education	No school	170 (88.1)	23 (11.9)	193 (57.8)
	Primary	3 (100)	0 (0.0)	3 (0.9)
	Quranic/Dugsi	129 (93.5)	9 (6.5)	138 (41.3)
Level of father's education	No school	284 (89.9)	32 (10.1)	316 (94.6)
	Primary school	11 (100)	0 (0.0)	11 (3.3)
	Secondary school	7 (100)	0 (0.0)	7 (2.1)
Level of mother's education	No school	289 (90.0)	32 (10.0)	321 (96.1)
	Primary school	12 (100)	0 (0.0)	12 (3.6)
	Secondary school	1 (100)	0 (0.0)	1 (0.3)
Father's occupation	Businessman	29 (85.3)	5 (14.7)	34 (10.2)
	Farmer	30 (96.8)	1 (3.2)	31 (9.3)
	Laborer	31 (88.6)	4 (11.4)	35 (10.3)
	Teacher	54 (96.4)	2 (3.6)	56 (16.8)
	Unemployed/other	158 (88.8)	20 (11.2)	178 (53.4)
Mother's occupation	Business	40 (95.2)	2 (4.8)	42 (12.6)
	Unemployed	262 (89.7)	30 (10.3)	292 (87.4)
Family size	0–5	129 (94.2)	8 (5.8)	137 (41.0)
	>5	173 (87.8)	24 (12.2)	197 (59.0)
Monthly family income (US${\$}$)	<100	240 (90.6)	25 (9.4)	265 (79.3)
	100–500	62 (89.9)	7 (10.1)	69 (20.7)
Duration of stay in camp	0–3 y (1–36 mo)	299 (90.9)	30 (9.1)	329 (98.5)
	>4 y (>37 mo)	3 (60.0)	2 (40.0)	7 (1.5)

### Household behavioral and environmental risk factors

Almost all the children (329; 98.5%) had no history of deworming. The results of the other potential risk factors revealed that 94.6% were in the habit of swimming in water and that 82.9% keep livestock in their houses. Sanitary habits indicate that 222 (66.5%), 276 (82.6%) and 214 (64.1%) wash their hands after defecation, wash their hands before and after eating, and eat only washed fruits, respectively (Table 2).

**Table 2.** Behavioral and environmental related risk factors related to Giardia infection

		Status of Giardia infection		
Variable	Category	Negative (%)	Positive (%)	Total (%)
Keeping of animals	Yes	50 (87.7)	7 (12.3)	57 (17.1)
	No	252 (91.0)	25 (9.0)	277 (82.9)
History of deworming	Yes	5 (100)	0 (0.0)	5 (1.5)
	No	297 (90.3)	32 (9.7)	329 (98.5)
Child swimming in water	Yes	17 (94.4)	1 (5.6)	18 (5.4)
	No	285 (90.2)	31 (9.8)	316 (94.6)
Washing hands after defecation	Yes	193 (86.9)	29 (13.1)	222 (66.5)
	No	109 (97.3)	3 (2.7)	112 (33.5)
Washing hands before and after eating	Yes	246 (89.1)	30 (10.9)	276 (82.6)
	No	56 (96.6)	2 (3.4)	58 (17.4)
Handwashing after soil contact	Yes	100 (90.9)	10 (9.1)	110 (32.9)
	No	202 (90.2)	22 (9.8)	224 (67.1)
Dirty fingernails	Yes	85 (95.5)	4 (4.5)	89 (26.6)
	No	217 (88.6)	28 (11.4)	245 (73.4)
Eating unwashed fruits	Yes	104 (86.7)	16 (13.3)	120 (35.9)
	No	198 (92.8)	16 (7.2)	214 (64.1)
Wearing footwear	Yes	174 (88.8)	22 (11.2)	196 (58.7)
	No	128 (92.8)	10 (7.2)	138 (41.3)
Sources of drinking water	Municipal tap water	172 (93.5)	12 (6.5)	184 (55.1)
	Water trucking	127 (86.4)	20 (13.6)	147 (44.0)
	Well water	3 (100)	0 (0.0)	3 (0.9)
Water storage	Plastic containers	30 (85.7)	5 (14.3)	35 (10.5)
	Tanks	272 (91.0)	27 (9.0)	299 (89.5)
Coverage of water container	Covered	195 (88.6)	25 (11.4)	220 (65.9)
	Uncovered	107 (93.9)	7 (6.1)	114 (34.1)
Sanitary condition at home and outside	Good	162 (89.0)	20 (11.0)	182 (54.5)
	Poor	140 (92.1)	12 (7.9)	152 (45.5)
Type of toilet	Open defecation	276 (89.6)	32 (10.4)	308 (92.2)
	Pit latrine	2 (100)	0 (0.0)	2 (0.6)
	Others	24 (100)	0 (0.0)	24 (7.2)
Sewage disposal	Dustbin	8 (100)	0 (0.0)	8 (2.4)
	Garbage pit	127 (97.7)	3 (2.3)	130 (38.9)
	Open field outside compound	123 (80.9)	29 (9.1)	152 (45.5)
	Other	44 (100)	0 (0.0)	44 (13.2)

### Logistic repression analysis of sociodemographic factors of *G. lamblia* infection

The bivariate and multivariate analysis revealed that age, place of residence and family size were statistically significant. Also, children aged <5 y (p=0.002), households in the Kahda IDP camp (p=0.019) and families with >5 members in their households (p=0.034) all have a higher risk of becoming infected with Giardia (Table 3).

**Table 3.** Bivariate and multivariate analysis of participants’ demographics with Giardia infection

		Status of Giardia infection					
Variable	Category	Negative	Positive	COR (95% CI)	p	AOR (95% CI)	p
Gender	Female	164	18	1		-	-
	Male	138	14	0.924 (0.444–1.926)	0.834	-	-
Age category	<5 y	176	29	1		1	
	6–10 y	109	3	0.167 (0.050–0.562)	0.004	0.157 (0.045–0.552)	0.004^*^
	11–15 y	17	0	0.000 (0.000–0.000)	0.998	0.000 (0.000–0.000)	0.998
Residence	Deyniile	140	21	1		1	
	Kahda	162	11	0.453 (0.211–0.972)	0.042	0.347 (0.151–0.798)	0.013^*^
Level of child’s education	No school	170	23	1		-	-
	Primary	3	0	0.000 (0.000–0.000)	0.999	-	-
	Quranic/Dugsi	129	9	0.516 (0.231–1.152)	1.106	-	-
Level of father's education	No school	284	32	1.820 (0.000–2.370)	0.999	-	-
	Primary school	11	0	1.000 (0.000–1.000)	1.000	-	-
	Secondary school	7	0	1		-	-
Level of mother's education	No school	289	32	1.788 (0.000–5.920)	1.000	-	-
	Primary school	12	0	1.000 (0.000–1.000)	1.000	-	-
	Secondary school	1	0	1		-	-
Father's occupation	Businessman	29	5	1		1	
	Farmer	30	1	0.193 (0.021–1.757)	0.144	0.199 (0.019–2.026)	0.173
	Laborer	31	4	0.748 (0.183–3.062)	0.687	1.196 (0.246–5.806)	0.824
	Teacher	54	2	0.215 (0.039–1.177)	0.076	0.464 (0.074–2.928)	0.414
	Unemployed/other	158	20	0.734 (0.255–2.113)	0.567	0.979 (0.292–3.277)	0.972
Mother's occupation	Business	40	2	1		-	-
	Unemployed	262	30	2.290 (0.527–9.955)	0.269	-	-
Family size	0–5	129	8	0.447 (0.195–1.027)	0.058	0.274 (0.111–0.676)	0.005^*^
	>5	173	24	1		1	
Monthly family income (US${\$}$)	<100	240	25	0.923 (0.381–2.232)	0.858	-	-
	100–500	62	7	1		-	-
Duration of stay in camp	0–3 y (1–36 mo)	299	30	0.151 (0.024–0.936)	0.042	0.337 (0.041–2.753)	0.310
	>4 y (≥37 mo)	3	2	1		1	

Abbreviations: AOR, adjusted OR; COR, crude OR.

*indicates statistical significance (p<0.05).

### Bivariate and multivariate analysis of behavioral and environmental factors of Giardia infection

This study evaluated various behavioral and environmental risk factors that predispose children to Giardia infection and tried to understand their relationship. Although there appears to be a considerable association between Giardia infection and these risk factors, none was found to be statistically significant (Table 4).

**Table 4.** Behavioral and environmental risk factors related to Giardia infection

		Status of Giardia infection					
Variable	Category	Negative	Positive	COR (95% CI)	p	AOR (95% CI)	p
Keeping of animals	Yes	50	7	1.411 (0.579–3.441)	0.449	-	-
	No	252	25	1		-	-
History of deworming	Yes	5	0	0.000 (0.000–3.910)	0.999	-	-
	No	297	32	1		-	-
Child swimming in water	Yes	17	1	0.541 (0.070–4.203)	0.557	-	-
	No	285	31	1		-	-
Washing hands after defecation	Yes	193	29	5.459 (1.625–18.338)	0.006	4.892 (0.904–26.473)	0.065
	No	109	3	1		1	
Washing hands before and after eating	Yes	246	30	3.415 (0.793–14.710)	0.099	0.889 (0.130–6.065)	0.904
	No	56	2	1		1	
Handwashing after soil contact	Yes	100	10	0.918 (0.419–2.013)	0.831	-	
	No	202	22	1			
Dirty fingernails	Yes	85	4	0.365 (0.124–1.071)	0.066	0.501 (0.122–2.050)	0.336
	No	217	28	1		1	
Eating unwashed fruits	Yes	104	16	1.094 (0.915–3.960)	0.085	2.941 (1.258–6.871)	0.013^*^
	No	198	16	1		1	
Wearing footwear	Yes	174	22	1.618 (0.741–3.536)	0.227	1.522 (0.478–4.848)	0.477
	No	128	10	1		1	
Sources of drinking water	Municipal tap water	172	12	1		1	
	Water trucking	127	20	2.257 (1.065–4.786)	0.034	2.074 (0.939–4.579)	0.071
	Well water	3	0	0.000 (0.000–1.000)	0.999	0.000 (0.000–1.000)	0.999
Water storage	Plastic containers	30	5	1		-	-
	Tanks	272	27	0.596 (0.213–1.662)	0.322		
Coverage of water container	Covered	195	25	1		1	
	Uncovered	107	7	0.510 (0.214–1.219)	0.130	1.395 (0.395–4.922)	0.605
Sanitary condition at home and outside	Good	162	20	1		-	-
	Poor	140	12	0.694 (0.328–1.471)	0.341	-	-
Type of toilet	Open defecation	276	32	1		-	-
	Pit latrine	2	0	0.000 (0.000–1.000)	0.999	-	-
	Others	24	0	0.000 (0.000–1.000)	0.998	-	-
Sewage disposal	Dustbin	8	0	1		-	-
	Garbage pit	127	3	3.816 (0.000–7.391)	0.999	-	-
	Open field outside compound	123	29	3.808 (0.000–6.927)	0.999	-	-
	Others	44	0	1.000 (0.000–4.904)	1.000	-	-

Abbreviations: AOR, adjusted OR; COR, crude OR.

*indicates statistical significance (p<0.05).

## Discussion

Findings from the current study showed that the overall prevalence of *G. lamblia* infection among children living in IDP settlements in Mogadishu, Somalia, was 9.6%. The results are higher than the 16% reported among children in rural Somalia, as well as 3.6% and 22.1% recently published in a 6-y retrospective study looking at the prevalence of intestinal parasites among patients at some selected hospitals in Somalia, and among malnourished children aged 6–59 mo, respectively.^9,10^ While in another related 5-y retrospective study of intestinal parasites in a tertiary hospital in Somalia, 60.84% prevalence of Giardia was reported.^11^ In each of the above studies, Giardia was found to have the highest prevalence rate, except for the study on malnourished children, where *Ascaris lumbricoides* had the highest rate with 46.6%, followed by *G. lamblia* with 22.1%.^10^ However, variabilities with our study could be because while we investigated children among the general population of IDP, the referenced studies evaluated sick patients diagnosed at the hospital. In the case of the malnourished children, the higher prevalence they reported may be because Giardia infection is more prevalent among malnourished children due to a combination of biological, environmental and socioeconomic factors that weaken their immune response, especially among displaced children that have increased exposure to contaminated food and water.^19^

Another study that was conducted in Pakistan among children aged <5 y found 3.31% prevalence, which was lower than in our study.^17^ Poor sanitation and hygiene, which are characteristic of IDP settlements in Somalia, create an environment where Giardia thrives, particularly in children.^2,20^ Also, contaminated water, food and surfaces increase exposure to the parasite, with children being more vulnerable due to frequent hand-to-mouth behaviors and underdeveloped immune systems, leading to higher infection rates and associated gastrointestinal illness.^2^

Among the sociodemographic characteristics of the participants studied, it was observed that relatively more females were infected by *G. lamblia* infection (18/32) than males (14/32); also, 23 children with no education were positive compared with nine and zero for children with Quranic education and primary education, respectively. However, no significant association was found between gender and the educational status of the children. This result is in contrast to studies conducted elsewhere, including Ethiopia and India, where the prevalence rate was higher among males than females.^4,15^ The male predominance as regards Giardia prevalence is attributed to the fact that male children have an increased tendency to be playful, including playing outdoors. In the case of the variation with the result of this investigation, it may be because the number of female children enrolled for the study was higher (164) than their male counterparts (138); it could also imply that gender may not be an important factor for giardiasis among children living in IDP settlements. This notion is supported by a study in southern Ethiopia among primary school students, where the prevalence rate was higher in female compared with male students.^21^

Other important sociodemographic characteristics evaluated include children's age, where we found that 90.6% of the children infected with Giardia belong to the <5 y age category, followed by 6–10 y (3.4%), while no children aged 11–15 y were found to be positive. Several studies have reported that giardiasis is more common in children aged <5 y due to their developing immune systems (which are often unable to clear mild infection), frequent hand-to-mouth activity and increased exposure to contaminated water or surfaces in communal settings like IDP settlements.^22–24^ Additionally, young children have poor hygiene practices, making them more susceptible to Giardia infections.^25^ This finding is consistent with studies conducted in Somalia, Ethiopia, Nigeria and Portugal.^10,23,26,27^

In terms of place of residence, it is interesting to note that, despite the number of children studied being higher in Kahda (173/334) compared with Deyniile (161/334) IDP camps, the result showed that the number of children infected with Giardia in Deyniile was almost double (21/32) the number of positive cases in Kahda (11/32), and this was significant. Deyniile and Kahda are two of the most populated IDP settlements in Somalia, and are predominantly occupied by people displaced by conflict and drought.^13^ One possible explanation could be that Deyniile has more verified IDP camps (1302), has more households (159 525) and hosts more individuals (739 137) compared with Kahda, which has 1108 IDP camps, 113 656 households and 607 938 individuals according to the 2022 Camp Coordination and Camp Management Cluster report.^28^

Another significant finding from this study is that families with a household size of >5 members had 75% (24/32) of the infections compared with families with <5 members, which had 15% (8/32) of the infections. Logistic regression analysis of this sociodemographic factor in relation to infections indicates that children from families with >5 members have more than twice as much risk of being infected by Giardia compared with children from families with <5 members. The result of this study is in agreement with a study among indigenous communities in Somalia, where it was found that participants from families with >7 members living together experienced a significantly higher prevalence of Giardia infection compared with those from smaller families.^20^ Larger households are at a higher risk of helminth infections due to a combination of factors such as increased person-to-person contact, overcrowding, poor sanitation, shared resources and limited access to healthcare.^16,20^ These conditions create a favorable environment for helminths like Giardia to spread within the household and persist in the community.

The findings of our study showed that all the children with *G. lamblia* infection belong to parents (mothers and fathers) who do not have any formal or informal education. Although no significant association was found upon multivariate logistic regression analysis, this finding implies that having educated parents could reduce the risk of infection, as demonstrated in a study from Portugal that observed the risk of *G. lamblia* infection as higher among children from uneducated mothers.^23^ The reason for this might be that educated parents are generally more aware of hygiene practices, water sanitation and disease prevention, which will invariably reduce the risk of exposure to Giardia in their households.^23,25^ Similarly, low-income families (earning <US${\$}$100 monthly) were also found to have higher cases of infection (25/32) compared with families earning US${\$}$100–500 monthly. Despite the fact this finding was not found to be significant in this study, it agrees with studies in Malaysia, India, Pakistan and Ethiopia, where economic status was reported to be significantly associated with Giardia infection.^4,17,20,26^ The lack of statistical significance with respect to participants’ economic status observed in this study could be attributed to the relatively small size of the reference group (US${\$}$100–500; n=62), which may limit the statistical power and the ability to detect significant differences.^29^ Additionally, the studied income brackets may not capture the full socioeconomic variability, particularly in a low-income setting that has experienced a prolonged period of humanitarian crisis where even those in the ‘higher’ category (i.e. US${\$}$100–500) may still experience poor living conditions.^30^

Some potential risk factors for *G. lamblia* infection were also investigated in this study, including behavioral (history of deworming, handwashing after defecation, handwashing before and after eating, eating unwashed fruits, wearing of footwear) and environmental (source of drinking water, water storage, open defecation, rearing of animals and sewage disposal) factors. Based on the results, none of the risk factors were found to be significantly associated with infection. Notwithstanding, the infection rates of *G. lamblia* were higher among children with no history of deworming, those who practice open defecation and those belonging to households that dispose of their waste in the open outside their houses.

Overall, our findings show that Giardia infection in Deyniile and Kahda IDP camps in Mogadishu was mostly associated with low socioeconomic status and poor WASH practices, coupled with a lack of education. From the general characteristics of both camps, it is clear that residents are predominantly uneducated, have a low monthly household income and unhealthy environmental sanitation habits, including open defecation and waste disposal in the open outside their homes. Hence, the government and other humanitarian actors with a presence in these camps should consider implementing lifestyle interventions in order to significantly lower the prevalence of Giardia and other helminth infections in their settlements.

### Study strengths and limitations

In this study, only a single fecal sample was collected, rather than the recommended three consecutive samples. This was because of financial constraints, as well as the difficulty in accessing the population due to security concerns. Notwithstanding, the findings of this study serve as a pointer for necessary public health intervention and can be generalized to other IDP settlements in Somalia. Also, the relatively small sample size used in this study due to the high security risk may have resulted in the lack of a statistically significant association between exposure and the outcome variables analyzed.

On the other hand, while previous studies in Somalia have focused on hospital-based and retrospective data on intestinal parasites, this research provides real-time, community-based prevalence data on Giardia infection among vulnerable displaced children. Similarly, by focusing on the Deyniile and Kahda IDP camps, the study provides insights into a high-risk, underserved population that has not been comprehensively studied. Lastly, the study systematically evaluates WASH behaviors in IDP camps and their direct correlation with Giardia infection. This detailed household behavioral and environmental assessment is unique and provides actionable insights for policy interventions.

### Conclusion

The outcome of this investigation shows that Giardia is prevalent in the IDP communities, and this constitutes a significant public health concern, particularly among the large, displaced populations in Somalia. The prevalence was found to be higher in the Deyniile IDP camp, in comparison with the Kahda IDP camp, which had a higher number of enrolled children. The prevalence was also found to be higher among children aged <5 y, as well as among children whose parents were uneducated and had a low monthly income, in addition to those living in poor sanitation conditions with poor personal hygiene. Addressing this public health problem would require commitment from both residents by changing their habits and embracing good hygiene behavior, as well as the government and other stakeholders by providing health education on good personal hygiene and good sanitary practices, as well as general awareness about soil and intestinal helminth infections. Targeted interventions by the government and humanitarian actors, such as improved WASH programs that would promote access to clean water sources, regular handwashing practices and proper sanitation infrastructure to reduce transmission within households, especially those of a large family size, would significantly help to address this important public health challenge. This study also recommends further longitudinal research to enable the understanding of seasonal prevalence. Also, evaluating the impact of targeted WASH interventions in IDP camps, as well as the antimicrobial resistance of Giardia, would provide more insights on how best to manage the problem. Hence, the results of the current study could provide baseline data within which this future research could be undertaken, especially because the camps we studied are the most overcrowded in Somalia.

## Data Availability

All data related to this article are contained within the article.
